# Empowering health personnel for decentralized health planning in India: The Public Health Resource Network

**DOI:** 10.1186/1478-4491-7-57

**Published:** 2009-07-20

**Authors:** Anuska Kalita, Sarover Zaidi, Vandana Prasad, VR Raman

**Affiliations:** 1 ICICI Centre for Child Health and Nutrition Centre, Pune, India; 2 Public Health Resource Centre, Delhi, India; 3 State Health Resource Centre, Raipur, India

## Abstract

The Public Health Resource Network is an innovative distance-learning course in training, motivating, empowering and building a network of health personnel from government and civil society groups. Its aim is to build human resource capacity for strengthening decentralized health planning, especially at the district level, to improve accountability of health systems, elicit community participation for health, ensure equitable and accessible health facilities and to bring about convergence in programmes and services.

The question confronting health systems in India is how best to reform, revitalize and resource primary health systems to deliver different levels of service aligned to local realities, ensuring universal coverage, equitable access, efficiency and effectiveness, through an empowered cadre of health personnel. To achieve these outcomes it is essential that health planning be decentralized. Districts vary widely according to the specific needs of their population, and even more so in terms of existing interventions and available resources. Strategies, therefore, have to be district-specific, not only because health needs vary, but also because people's perceptions and capacities to intervene and implement programmes vary. In centrally designed plans there is little scope for such adaptation and contextualization, and hence decentralized planning becomes crucial.

To undertake these initiatives, there is a strong need for trained, motivated, empowered and networked health personnel. It is precisely at this level that a lack of technical knowledge and skills and the absence of a supportive network or adequate educational opportunities impede personnel from making improvements. The absence of in-service training and of training curricula that reflect field realities also adds to this, discouraging health workers from pursuing effective strategies.

The Public Health Resource Network is thus an attempt to reach out to motivated though often isolated health workers. It interacts with, and works to empower, health personnel within the government health system as well as civil society, to meaningfully participate in and strengthen decentralized planning processes and outcomes. Structured as an innovative distance-learning course spread over 12 to 18 months of coursework and contact programmes, the Public Health Resource Network comprises 14 core modules and five optional courses. The technical content and contact programmes have been specifically developed to build perspectives and technical knowledge of participants and provide them with a variety of options that can be immediately put into practice within their work environments and everyday roles. The thematic areas of the course modules range from technical knowledge related to maternal and child health and communicable and noncommunicable diseases; programmatic and systemic knowledge related to health planning, convergence, health management and public-private partnerships; to perspective-building knowledge related to mainstreaming gender issues and community participation. Currently the Public Health Resource Network has been launched in four states of India – Chhattisgarh, Jharkhand, Bihar and Orissa – in its first phase, and reaches out to more than 500 participants with diverse backgrounds. The initiative has received valuable support from central and state government departments of health, state training institutes, the National Rural Health Mission – the current comprehensive health policy in the country – and leading civil society organizations.

## Introduction

### Rationale and scope

The question confronting health systems in India is how best to reform, revitalize and resource primary health systems to deliver different levels of service aligned to local realities, ensuring universal coverage, equitable access, efficiency and effectiveness, through an empowered cadre of health personnel. One of the important prerequisites to achieving these outcomes is decentralized health planning to include conceptualization and operationalization of health programmes at local levels, as well as decentralized governance of systems of planning and delivery, at least at the level of the district. In India, a district is the smallest administrative unit. The country has 604 districts across its 28 states and seven Union Territories.

The district is considered the most appropriate level for operationalizing primary health. It is the basic unit of development, where agencies of various social development sectors, including health, plan and implement their programmes, thus providing a unique opportunity for integration and intersectoral coordination. The district provides an opportunity for interface between policy and implementation of health programmes at the level of the community, in addition to being a composite unit of the health system with a clearly defined administrative and geographical area – including the health subcentre (for 3000 to 5000 persons), the primary health centre (30 000 to 50 000 persons), the community health centre (80 000 to 100 000 persons), and the district hospital (catering to a population of about one million).

Districts vary widely according to the specific needs of their population, and even more so in terms of existing interventions and available resources. Strategies, therefore, must be district-specific, not only because health needs vary, but also because people's perceptions and capacities to intervene and implement programmes vary. In centrally designed plans there is little scope for such adaptation and contextualization, hence decentralized planning becomes crucial [1].

District health plans have also assumed a new centrality and urgency in the current context of the National Rural Health Mission (NRHM), 2005–2012, which was announced in April 2005 by the Government of India with the stated goal "to promote equity, efficiency, quality and accountability of public health services through community driven approaches, decentralisation and improving local governance". The NRHM includes decentralization of health planning to empower local governments to manage, control and be accountable for public health services as a core strategy [2].

### The challenges

For such planning to take place effectively, there is a strong need for trained, motivated, empowered and networked health personnel. But it is at this level that a lack of technical knowledge and skills and the absence of a supportive network or adequate educational opportunities impede personnel from making improvements. The limited nature of in-service training and of training curricula that reflect field realities add to this, discouraging health workers from pursuing effective strategies. There is also the need to evolve from a more "command and control" orientation of public health officials towards the community, to an attitude of participation, openness and accountability, recognizing the rights of the poor and the vulnerable. Capacity building is also needed in civil society groups, for members who are active in forums such as District Health Societies, district planning teams, hospital management committees and in the implementation of community health programmes.

One of the major gaps repeatedly identified by public health experts in the capacity of public health officials is the lack of experience and perspectives in the socioeconomic, cultural and political aspects of health and poverty [3]. Lack of capacity to analyse and interpret "what is really going on" in their area has led to an absence of district health planning and consequent outsourcing of this exercise to international technical assistance groups. This only propagates the situation of apathy and non-ownership on the part of the health officials.

These gaps must be addressed systematically in order to bring about the desired achievements in decentralized planning. The Public Health Resource Network is an effort towards this end.

## Discussion

### The Public Health Resource Network

Started in 2005, the Public Health Resource Network (PHRN) is an innovative distance-learning course in training, motivating, empowering and building a network of existing health personnel from government and civil society groups. Its aim is to build human resource capacities for strengthening decentralized health planning and to reach out to motivated, though often isolated, health workers. Thus, PHRN's objectives are as follows:

• reaching out to dedicated individuals to whom health equity is a major concern, and giving them access to essential information and opportunities to contribute to this goal;

• sharing public health technical resources with existing and potential district health programme managers towards strengthening the public health system in their districts, and assisting in the emergence of state and district resource groups for this purpose;

• empowering civil society to create spaces, and using the spaces being created under the NRHM, to improve and increase public participation in health planning and management;

• promoting decentralization and horizontal integration at district, block and village levels by building capacity in technical, programmatic, epidemiological and social understandings of health;

• strengthening the resource base needed for informed advocacy within the government and civil society;

• facilitating networking and mutual support among public health practitioners.

Structured as an innovative distance-learning course spread over 12 to 18 months of coursework and contact programmes, the PHRN comprises 14 core modules and five optional courses. The technical content and contact programmes have been specifically developed to build perspectives and technical knowledge of participants and provide them with a variety of options that can be immediately put into practice within their work-environment roles. The thematic areas of the course range from technical knowledge related to maternal and child health, communicable and noncommunicable diseases; programmatic and systemic knowledge related to health planning, convergence, health management, and public-private partnerships; to perspective-building knowledge related to mainstreaming gender issues and community participation.

More specifically, the course covers the following main themes: Quarter 1 – Introduction to Public Health Systems; Reduction of Maternal Mortality; Accelerating Child Survival; Community Participation and Community Health Workers; Behaviour Change Communication and Training. Quarter 2 – Mainstreaming Women's Health Concerns; Community Participation; Disease Control Programmes; Convergence; District Health Planning. Quarter 3 – District Health Management; Public-Private Partnership; Legal Framework of Health Care; Issues of Governance and Health Sector Reform. Quarter 4 (Optional Courses) – Tribal Health; Urban Health; Hospital Administration;

Noncommunicable Diseases and Mental Health; Disaster and Epidemic Management. The PHRN now operates in the states of Chhattisgarh, Jharkhand, Bihar and Orissa, with more than 500 participants. Initially supported by the State Health Resource Centre Chhattisgarh (SHRC), the PHRN is currently coordinated by the Public Health Resource Society (PHRS), which provides continuous support to the four state offices. The initiative has received valuable support from the NRHM at both central and state levels from state training institutes, the National Health Systems Resource Centre (NHSRC), and leading civil society organizations, including the Child In Need Institute (CINI), the Population Foundation of India (PFI) and the ICICI Centre for Child Health and Nutrition (ICCHN).

Besides the regular course, one other strategy of the PHRN is the fast-track capacity-building programme that is organized in collaboration with state governments willing to invest in their human resources. Such fast-track programmes have been organized in collaboration with the state governments in Arunachal Pradesh, Assam, Chhattisgarh, Manipur, Meghalaya, Mizoram, Nagaland, Sikkim and Tripura. Constructed as three rounds of a six-day-long training workshop held three to four months apart, this is focused on capacity building of government personnel working with NRHM for district-level planning. The goal is to build adequate skills in a team of about five resource persons per district for the next five years to create a pool of 25 public health officials from among motivated individuals from the government, from which a district resource unit can be made functional, to facilitate district health plans of good quality based on situational analyses, and to develop capacity to train *panchayat *(lowest unit of decentralized governance) officials and civil society groups in effective outcome-oriented village health planning [4].

Implementing the PHRN has contributed to valuable experience for establishing and sustaining a people's network, in close collaboration with the government, towards the end objective of building capacity to improve decentralized planning and programming. In terms of course participants, while the response to enrolment, contact sessions and the use of course material have been very encouraging, the challenge has been to motivate participants to undertake projects and assignments, especially due to the absence of any current formal accreditation. The collaboration of the government in organizing the fast-track capacity-building programmes and participation of the health system personnel have been positive, although follow-up of these concentrated sessions for translation into policy and practice must be strengthened. The cooperation of individual resource persons in volunteering for contact sessions and fast-track capacity-building programmes has made the decentralized operationalization of the PHRN possible.

## Conclusion

PHRN, independent of its capacity-building role, also must promote all interventions that would improve NRHM outcomes. To attain these larger goals, the PHRN has expanded its scope.

Two new initiatives of the PHRN are:

1. accreditation through the Indira Gandhi National Open University (IGNOU) for a postgraduate diploma in district health management. Participants who enrol in the course through IGNOU and fulfill the stipulated credits on the basis of course assignments and evaluations would be awarded the diploma.

2. to create and support a fellowship programme. The fellows supported through this programme would be placed in district health societies and local civil society groups, with strong and continuous mentoring support from a network of resource individuals and organizations from across the country. The envisaged role of these fellows is to support all community-level processes in the districts through advocacy, appraisal of training and community processes, formative studies for designing community programmes and improving training curricula, and documentation of ongoing processes.

An effort towards improving the PHRN has been an exchange of experiential learning with the distance-learning course for a diploma/master's degree in public health offered by the School of Public Health (SOPH) at the University of the Western Cape in South Africa. Sharing of course material between the two programmes, interaction with the SOPH to share opportunities and challenges of implementation and future directions, and conceptualizing partnerships in research have been valuable in strengthening the PHRN and planning for its future trajectory.

The PHRN is thus a network that responds to the unique needs of changing realities. It is an effort to build capacity and empower the participants to translate knowledge into action, and to bring about positive, equitable and sustainable change.

## Competing interests

The authors declare that they have no competing interests.

## Authors' contributions

AK conceptualized the structure of the manuscript; AK, VP, SZ and VRR worked on the manuscript. All the authors read and approved the final manuscript.
